# Supplementary material for: Broussochalcone A Is a Novel Inhibitor of the Orphan Nuclear Receptor NR4A1 and Induces Apoptosis in Pancreatic Cancer Cells
Source: Molecules. 2021 Apr 16;26(8):2316. doi: 10.3390/molecules26082316 (PMC8073833; doi:10.3390/molecules26082316)
Supplement: Supplementary file 1 [file molecules-26-02316-s001.zip › molecules-1183092-supplementary.pdf]

SUPPLEMENTAL METERIALS

1 Table

3 Figures

Table S1. Chromatographic conditions for HPLC

Instrument	LC Condition
Column	Phenomenex Kinetex C18<br>(150 mm × 4.6 mm × 2.6 μm, 100A)
Injection volume	5 μL (1 mg/mL)
Flow rate	1 mL/min
Detector	ELSD
Column temperature	20°C
Mobile phase/Time (min)	2% B (0 min) → 100% B (20 min)
	Gradient for 30 min

A: water containing 0.1% trifluoroacetic acid

B: acetonitrile containing 0.1% trifluoroacetic acid

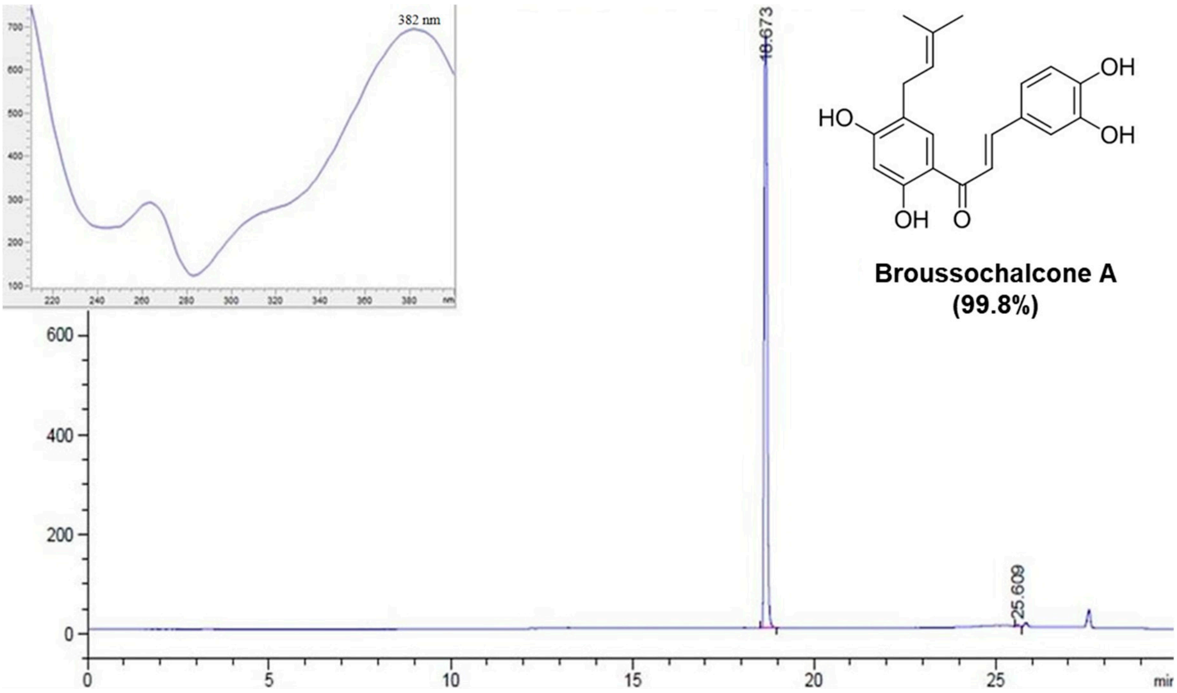

Figure S1. The UV-vis absorption, HPLC spectrum and chemical structure of BCA isolated from *B. papyrifera* roots.

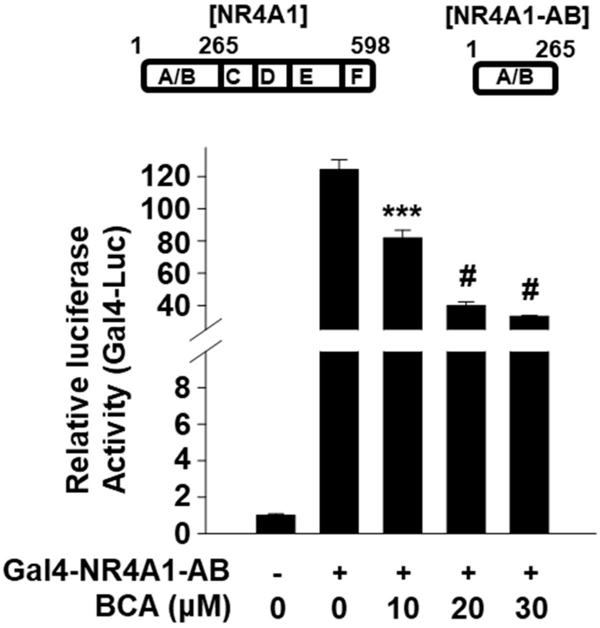

Figure S2. Effect of BCA on luciferase activity in MiaPaCa-2 cells transfected with Gal4-RE-Luc/C-terminal deletion Gal4-NR4A1 chimera (Gal4-NR4A1-AB). Cells were cotransfected with Gal4-RE-Luc (25 ng) and 5 ng of Gal4-NR4A1-AB for 5 h, and then treated with BCA for 18 h. Luciferase activity (relative to β-galactosidase) was determined, and the corresponding empty vector was used as a control. The results are presented as means ± SEM. ****P*<0.005 and $^{\#}$*P*<0.001 vs. DMSO + Gal4-NR4A1-AB.

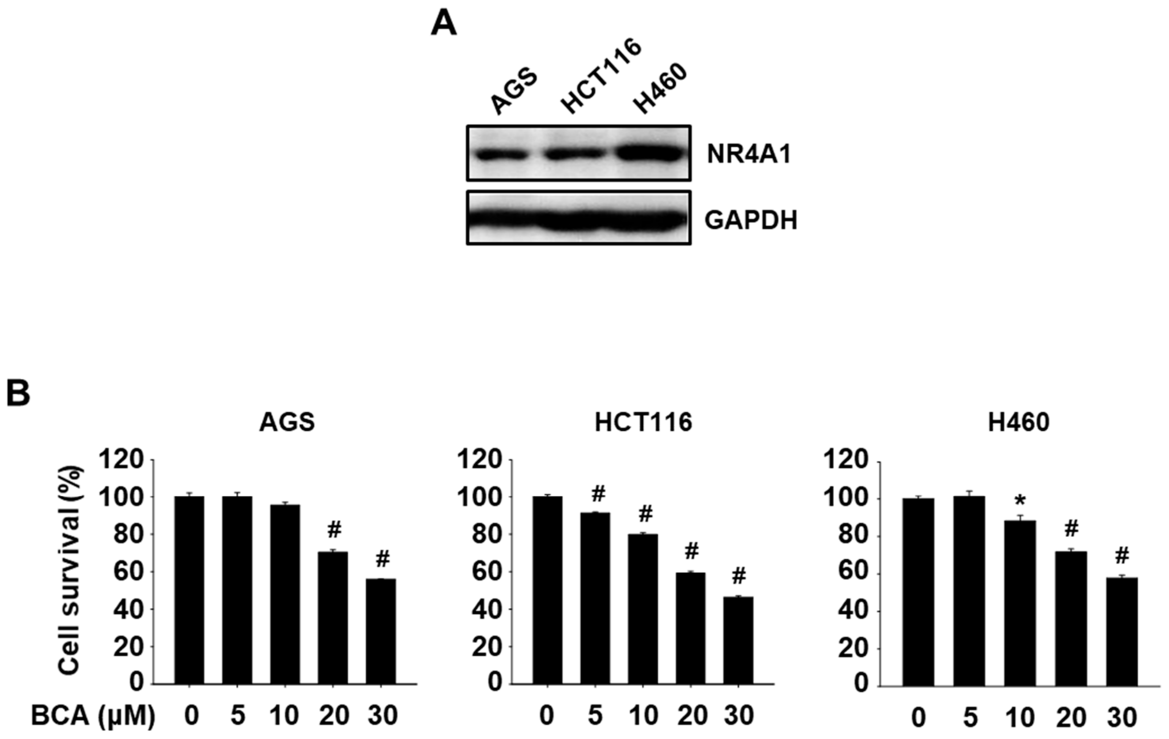

Figure S3. Effects of BCA on cell growth in various cancer cell lines expressing NR4A1. (A) Whole cell lysates of each cell lines were analyzed by western blot analysis. GAPDH was used as a loading control. (B) Cells were treated with BCA for 24 h, and cell viability was determined as described in the Materials and Methods. The results are presented as means ± SEM. **P*<0.05 and #*P*<0.001 vs. DMSO.
